# Structuring higher-order thinking: a national analysis of learning outcomes in Swedish undergraduate nursing thesis courses

**DOI:** 10.1186/s12912-025-03824-0

**Published:** 2025-09-01

**Authors:** Martin Salzmann-Erikson, Henrik Eriksson, Ove Björklund, Åsa Hedlund

**Affiliations:** 1 Department of Caring Sciences, Faculty of Health and Occupational Studies, University of Gävle, Gävle, SE-801 76 Sweden; 2 Department of Health Sciences, University West, Trollhättan, Sweden

**Keywords:** Undergraduate, Nursing education, Thesis, Academic writing, Blooms taxonomy, Learning outcomes, National analysis, Sweden

## Abstract

**Background:**

Higher-order thinking is a central objective in nursing education, particularly within thesis courses where students are expected to demonstrate analytical reasoning and scholarly autonomy.

**Aim:**

The aim of this study is to examine the structure, cognitive complexity, and knowledge domain classification of learning outcomes in degree project courses within Swedish undergraduate nursing education.

**Methods:**

This national cross-sectional study examined the cognitive structure of 236 intended learning outcomes derived from 23 universities and university colleagues offering undergraduate nursing thesis courses across all Swedish higher education institutions (*N* = 25). Active verbs were extracted and analyzed using manifest content analysis, descriptive statistics, Mann–Whitney U tests, and Spearman’s rank correlation.

**Results:**

Using Bloom’s revised taxonomy as the analytical framework, we identified 58 unique active verbs. All institutions included outcomes at multiple taxonomic levels, with “Analyzing” and “Applying” most frequently used. In contrast, “Understanding” was rarely represented, despite its foundational role in cognitive progression. Lexical diversity and alignment with higher-order thinking varied significantly across institutions. One-third of the verbs were not included in Bloom’s taxonomy, highlighting the interpretive challenges in applying taxonomic models to curriculum analysis.

**Conclusion:**

These findings suggest divergent pedagogical assumptions underlying outcome design and underscore the need for more coherent, epistemologically informed approaches to ensure thesis courses truly support academic development. The results may inform quality assurance practices and contribute to ongoing debates about the role of research training in undergraduate nursing education.

## Introduction

Higher-order thinking has long been recognized as a cornerstone of educational theory and curriculum development, particularly following the introduction of Bloom’s taxonomy of educational objectives [1–4]. Bloom’s original taxonomy positioned cognitive processes along a continuum, from basic recall to complex tasks such as evaluation and creation. In the revised version by Anderson and Krathwohl [5], the categories of Analyze, Evaluate, and Create are understood as constituting higher-order cognitive processes—those that demand not only engagement with knowledge but its critical appraisal, reorganization, and innovative application. These capabilities are not merely academic ideals but essential competences for professions such as nursing, where clinical reasoning, ethical judgment, and decision-making under uncertainty are everyday requirements [5–7]. However, as Säfström [8] points out, developing such capacities involves more than accumulating information; it demands a qualitative deepening of epistemic engagement. Without deliberate curricular design, aspirations toward higher-order thinking risk becoming rhetorical rather than transformative.

While foundational skills like remembering, understanding, and applying provide an essential base, they are insufficient for cultivating the adaptive expertise increasingly demanded in professional practice [9]. Despite widespread emphasis on research-related learning goals, curricular structures often continue to privilege procedural knowledge at the expense of critical inquiry and intellectual curiosity [10], reflecting a persistent instrumentalism in educational practice.

Within nursing education, recent studies have repositioned the thesis course as more than a procedural requirement [11–14]. Henttonen and colleagues have illuminated how students in Swedish nursing programs engage with thesis writing as a process of identity formation, epistemic negotiation, and practical integration. Their work highlights that students do not merely write “about” nursing—they write themselves *into* the profession, often grappling with tensions between abstract academic expectations and the concrete, situated realities of clinical practice. The thesis thus becomes a mediating artefact between the university and the healthcare field: a site where students rehearse critical reasoning, navigate ethical ambiguities, and articulate their emerging professional voices. However, how higher-order thinking is integrated in the thesis by examining learning goals and active verbs has not previously been investigated. An active verb explicitly states the cognitive action required of the student, for example *to compare*, or *to analyze.* This transformative potential is echoed in findings by Dogan et al. [15] who show that bachelor’s thesis work fosters enduring positive attitudes toward research and evidence-based practice. Yet the nature of thesis work is shaped by methodological traditions; as Roca et al. note, nursing thesis overwhelmingly favor qualitative, descriptive approaches, which may implicitly frame students as reflective practitioners rather than experimental researchers. Karlsholm et al. [16] emphasize further highlight the complexity of the thesis experience, documenting the strategies students employ to navigate both cognitive and emotional demands.

Despite this growing body of research of nursing pedagogy relates to higher-order thinking to develop clinical skills [7, 17, 18], the interest for intended learning outcomes for thesis courses remains largely unexplored. While policy documents [19, 20] invoke analytical and critical thinking as desired outcomes, it is less clear whether these aspirations are systematically embedded at the curricular level. This gap is particularly significant given that learning outcomes serve not only as assessment tools but as signals of pedagogical priorities and cognitive ambitions.

Institutional structures also warrant attention. In Sweden, universities receive over 80% of public research block grants and employ approximately six times more full-time researchers than university colleges—12,000 versus 2,000 full-time equivalents—exposing enduring resource and status asymmetries [20]. Although newcomer colleges have sought to emulate university research norms—a process termed “academic drift” [21] it remains an open question whether such structural disparities translate into differences in the cognitive ambition of thesis course learning outcomes. Prior econometric analyses suggest that increased research and development investment correlates with enhanced scholarly output without compromising quality [22], but the curricular consequences of these structural differences have not been systematically investigated.

In Sweden all 25 accredited nursing programs culminate in a compulsory, stand-alone bachelor *thesis* worth 15 ECTS. Students first complete a separate 15-credit course in research methodology; at two institutions the research method course were merged with the thesis into a single 30-credit course, but the thesis portion still fulfils the 15-credit national requirement. Upon completion, nearly every student deposits the thesis in an open-access institutional repository (most commonly DiVA), making it publicly searchable and accessible in full-text, except for Karolinska Institute. Thus, the Swedish bachelor thesis functions as a fully-fledged research project, most often as a research-based literature review and completed together with a peer student [23].

Higher education policy frameworks add further complexity. The Swedish Higher Education Authority [24] explicitly identifies analytical and critical competencies as benchmarks for high-quality thesis work. Yet, as Säfström [8] and van Ooijen-van der Linden et al. [10] argue, policy declarations alone do not guarantee that these cognitive goals permeate curriculum design and pedagogical practice. A disconnect between aspiration and implementation remains a persistent risk.

Taken together, these strands reveal a two-fold problem. First, Swedish nursing programs must strike a credible balance among *epistēmē* (scientific knowing), *technē* (procedural skill) and *phronēsis* (situated judgement); yet anecdotal concerns about “over-academicization” suggest that technē may be crowding out the other two. Second, an emergent political debate exemplified by a political the party in Sweden idea to abolish the compulsory bachelor thesis as a cost and time-saving measure highlights how curricular decisions are now framed as solutions to workforce shortages rather than as vehicles for epistemic growth [25]. By mapping the cognitive demands already encoded in thesis-course outcomes, our study provides an empirical baseline for judging what would be lost—or must be re-engineered—should Sweden move towards a shorter, thesis-free nursing degree. Against this backdrop, the present study addresses an important gap by systematically examining how intended learning outcomes are formulated in undergraduate thesis courses across Swedish higher education institutions (HEIs) offering nursing programs. Using Bloom’s revised taxonomy [5] as an analytical framework, we (i) map the cognitive structure of each syllabus—that is, how its active verbs are distributed across Bloom’s six hierarchical categories—and (ii) assess domain alignment, defined as the degree to which every learning outcome is explicitly tied to one of Sweden’s three statutory knowledge domains: Knowledge and Understanding, Competence and Skills, or Judgement and Approach [19]. While learning outcomes ostensibly serve to articulate educational intent and guide student development, their actual cognitive demands—particularly at the critical juncture of thesis work—have yet to be fully mapped. Our analysis seeks to illuminate how curricular formulations encode expectations for professional reasoning, intellectual ambition, and knowledge production within the final stage of undergraduate nursing education.

## Objective and research questions

The aim of this study is to examine the structure, cognitive complexity, and knowledge domain classification of learning outcomes in degree project courses within Swedish undergraduate nursing education.

- How are the learning outcomes for degree project courses in nursing structured across Swedish HEIs, and to what extent are they aligned with Bloom’s revised taxonomy?
- What is the distribution and frequency of active verbs used in these learning outcomes, and how do these correspond to lower- and higher-order cognitive processes of the revised taxonomy?
- How are the learning outcomes distributed across the three national domains of knowledge (knowledge and understanding, competence and skills, judgement and approach), and how do these domain placements relate to their revised-Bloom coding?
- What correlations exist between domain classification and the use of higher-order cognitive active verbs (Analyze, Evaluate, Create) in Bloom’s hierarchy?
- Can distinct institutional patterns or typologies be identified in learning-outcome formulation when profiled with metrics derived from the revised Bloom taxonomy?

## Methods

### Design and methodological approach

This study employed a descriptive and analytical design, combining quantitative content extraction with descriptive and inferential statistics. Bloom’s revised taxonomy [5] served as the analytical framework to examine both the structural composition of course syllabi and the cognitive demands embedded in learning outcomes. The study focused on Bachelor’s degree project syllabi in nursing programs across Swedish HEIs.

### Setting and sample

The initial sampling frame comprised all 29 Swedish HEIs. Of these, 25 institutions offered a nursing program including a Bachelor’s thesis component. After excluding two institutions where the thesis was embedded within other courses, the final sample included 23 HEIs with publicly accessible syllabi (15 European Credit Transfer System [ECTS]) retrieved from institutional websites and official digital archives. In Sweden, nursing programs are required by the Higher Education Ordinance [26] to ensure that graduates achieve competencies across three national domains: knowledge and understanding, competence and skills, and judgment and approach. However, there is no formal requirement that course syllabi must organize learning outcomes explicitly according to these three domains. In our analysis, it became evident that a majority of institutions nonetheless structured their syllabi in this way, which enabled a domain-specific analysis of intended learning outcomes.

### Data collection and preparation

All intended learning outcomes were extracted verbatim from the syllabi and compiled into a dataset. Each outcome was examined to isolate active verbs—defined as verbs that explicitly express the cognitive action expected of students. An initial list of 70 distinct active verbs, expressed in Swedish, was generated. Semantic overlaps were identified and merged to avoid artificial inflation of conceptual diversity (e.g.,”kritiskt analysera” (Eng: “critically analyze”) and”analysera” (Eng: “analyze”) were treated as equivalent). Subsequently, all verbs were translated into English. Where Swedish variants converged into a single English term lacking cognitive distinction, further consolidation was performed (e.g., “rapportera” and”redovisa” were grouped under”report”). Coding decisions were collaboratively reviewed among co-authors to ensure analytical coherence and fidelity to Bloom’s taxonomy. After this two-stage process—semantic and linguistic—the final list comprised 58 unique active verbs, preserving institutional attribution for subsequent analyses.

### Data structuring of learning outcomes and taxonomic categorization

A structured dataset was established in Microsoft Excel, where each HEI constituted a single row and variables included institutional name, classification (university or university college), ETCS, and whether syllabi were organized according to the tripartite outcome structure defined in the Higher Education Ordinance (Table 1). Of the 23 syllabi, the total number of learning outcomes was 233; 17 (79.3%) syllabi explicitly adhered to this tripartite structure, yielding 172 categorized learning outcomes. The remaining six syllabi contained defined learning outcomes (*n* = 53) but lacked explicit categorization. This distinction was retained to inform subsequent subset analyses. The number of learning outcomes assigned to each national dimension was recorded, along with counts of total and unique active verbs per HEI. Each active verb was classified into one of Bloom’s six taxonomy categories—Remembering, Understanding, Applying, Analyzing, Evaluating, or Creating—through independent coding and peer-review consensus.

**Table 1:** Overview of included higher education institutions

Higher Education Institution	Kind of institution	Course name	ETCS	Structured syllabi
Blekinge Institute of Technology	University colleague	Degree Project in Nursing		15 YES
Dalarna University	University colleague	Thesis in Nursing Science		15 NO
Halmstad University	University colleague	Nursing - Thesis		15 YES
Jönköping University	University	Nursing Science, Thesis		15 YES
Karlstad University	University	Degree Project in Nursing		15 YES
Karolinska Institute	University	Degree Projet in Nursing		15 YES
Linköping University	University	Degree Project in Nursing Scien		15 YES
Linnaeus University	University	Degree project		15 NO
Luleå University of Technology	University	Nursing: Thesis		15 YES
Lund University	University	Bachelor Thesis in Nursing (Deg		15 YES
Marie Cederschiöld University College	University colleague	Independent Degree Project,		15 YES
Mid Sweden University	University	Degree Project		15 NO
Mälardalen University	University	Degree project in Care Science		15 NO
Red Cross University College	University colleague	Thesis in Nursing		15 YES
Sophiahemmet University College	University colleague	Thesis in Nursing Science		15 YES
Umeå University	University	Thesis for a Degree of Bachelor		15 YES
University of Borås	University colleague	Bachelor’s Degree Project in Car		15 YES
University of Gothenburg	University	Degree Project in Nursing		15 YES
University of Gävle	University colleague	Degree Thesis in Nursing Scienc		15 NO
University of Skövde	University colleague	Examensarbete i omvårdnad		15 YES
University West	University colleague	Degree project in Nursing, Bach		15 YES
Uppsala University	University	Thesis in Caring Sciences		15 YES
Örebro University	University	Nursing Science, Degree Project		15 NO

### Active verb frequency and cognitive structuring

The frequency of active verbs was calculated both overall and per HEI, with each verb categorized according to Bloom’s revised taxonomy. The distribution across the six cognitive levels–Remembering, Understanding, Applying, Analyzing, Evaluating, and Creating–was analyzed to assess the cognitive focus of the syllabi. In addition to total counts (*N* = 449), the number of unique verbs and the number of represented cognitive levels were recorded for each HEI. The proportion of higher-order cognitive verbs (Analyzing, Evaluating, Creating) was also calculated as a percentage of all active verbs, revealing variation in the emphasis on higher-order thinking skills across institutions (Table 3).

### Data analysis

Descriptive statistics were first used to summarize the number of learning outcomes (*N* = 233), total number of active verbs (*N* = 449), and total number of unique active verbs (*N* = 315) per syllabus (*N* = 23). Measures of central tendency (mean, median) and dispersion (standard deviation, range) were calculated manually in Excel to maintain transparency and allow for direct comparison between institutional data points, characterizing the variation across institutions. These descriptive metrics informed the interpretation of cognitive breadth and linguistic diversity in learning outcomes formulations.

To assess the relationship between the quantity of learning outcomes and the complexity of active verb usage, Spearman’s rank correlation coefficient (ρ) was calculated for the total number of learning outcomes in relation to both the total number of active verbs and the number of unique active verbs. As Excel lacks a dedicated function for Spearman’s ρ, a two-step approach was used: variables were first ranked manually using the RANK.AVG or RANK.EKV functions, after which the Pearson correlation formula (CORREL) was applied to the ranked data. In this way, correlation coefficients were computed using the Pearson method on ranked data, thereby yielding Spearman’s ρ. Exact p-values were calculated separately in Python to evaluate the statistical significance of observed associations.

To test group-level differences between universities and university colleges, non-parametric Mann–Whitney U tests were performed on the number of learning outcomes, total active verbs, and unique active verbs [27]. Given the small sample size and non-normal distribution of the data, this rank-based approach was deemed appropriate. Ranks were calculated manually in Excel and exact p-values were derived using *SciPy* [28] in Python.

For analyses involving the alignment of learning outcomes with the three domains defined in the Higher Education Ordinance, only the 17 syllabi with explicit structural categorization were included. This ensured internal consistency and allowed for domain-level proportional analysis, while preserving the full dataset for all other descriptive and correlational analyses.

For syllabi explicitly structured according to the three dimensions defined in the Swedish Higher Education Ordinance [19]—knowledge and understanding, competence and skills, and judgement and approach—a subset analysis was performed (*n* = 17). The number of learning outcomes in each category was recorded, and category-specific proportions were calculated relative to the total number of learning outcomes in each syllabus.

Furthermore, each syllabus was coded for the number of Bloom’s revised taxonomy [5] levels represented, providing a measure of cognitive breadth. The number of taxonomy levels (out of six possible) was then correlated with the proportion of higher-order active verbs to assess whether wider cognitive coverage coincided with increased complexity. The significance level was set at *p* < 0.05.

### Use of AI tools for statistical calculations and visualization

During data analysis, we used ChatGPT (versions 4o, 4.5, and o1) as a coding assistant in Google Colab to generate Python scripts for calculating exact p-values and creating visualizations of the results. All code was reviewed, edited, and executed by the authors; no statistical inference or interpretive output was accepted without verification of two co-authors. No generative AI images were created or used. This reporting aligns with Springer Nature’s guidance on documenting the use of large language models in research.

## Results

### Descriptive overview of learning outcomes and active verb usage

Our first research question concerned how the learning outcomes for degree project courses in nursing are structured across Swedish HEIs, and to what extent they are aligned with Bloom’s revised taxonomy. Our results showed that across the 23 included HEIs, the number of learning outcomes per syllabus ranged from 7 to 17, with a mean of 10 (SD = 2.28). The total number of active verbs per syllabus ranged from 12 to 31 (*M* = 19, SD = 4.78), and the number of unique active verbs ranged from 10 to 19 (*M* = 14, SD = 2.75). While total active verb counts capture the overall frequency of cognitive actions, the number of unique active verbs reflects lexical and pedagogical diversity—indicating the range of cognitive processes that syllabi aim to elicit. The proximity between mean and median values for learning outcomes suggests a relatively symmetric distribution, whereas greater variability in total and unique active verbs reflects heterogeneity in linguistic style and lexical richness. Spearman’s rank correlation coefficients (see Table 2) indicated a moderate positive association between the number of learning outcomes and the total number of active verbs (ρ = 0.61, *p* = 0.002), as well as a strong positive association with the number of unique active verbs (ρ = 0.69, *p* < 0.001).

**Table 2:** Spearman’s rank correlations between learning outcomes and verbs

Variable 1	Variable 2	Spearman’s ρ	*p*-value*
Learning outcomes	Total active verbs	0.61	0.002
Learning outcomes	Unique active verbs	0.69	< 0.001

Exact two-tailed p-values from t-approximation

### Institutional comparisons: universities vs university colleges

In our search for distinct institutional patterns or typologies, group comparisons between universities (*n* = 13) and university colleges (*n* = 10), conducted using the Mann–Whitney U test, revealed no statistically significant differences. The number of learning outcomes showed a non-significant trend (*U* = 37, *p* = 0.084, two-tailed), while no significant differences were observed for the total number of active verbs (*U* = 55, *p* = 0.552) or the number of unique active verbs (*U* = 47.5, *p* = 0.286).

### Cognitive complexity and higher-order active verbs

To investigate cognitive complexity, the proportion of active verbs classified within the higher-order categories of Bloom’s revised taxonomy (Analyzing, Evaluating, Creating) was calculated for each syllabus.

**Fig. 1:**
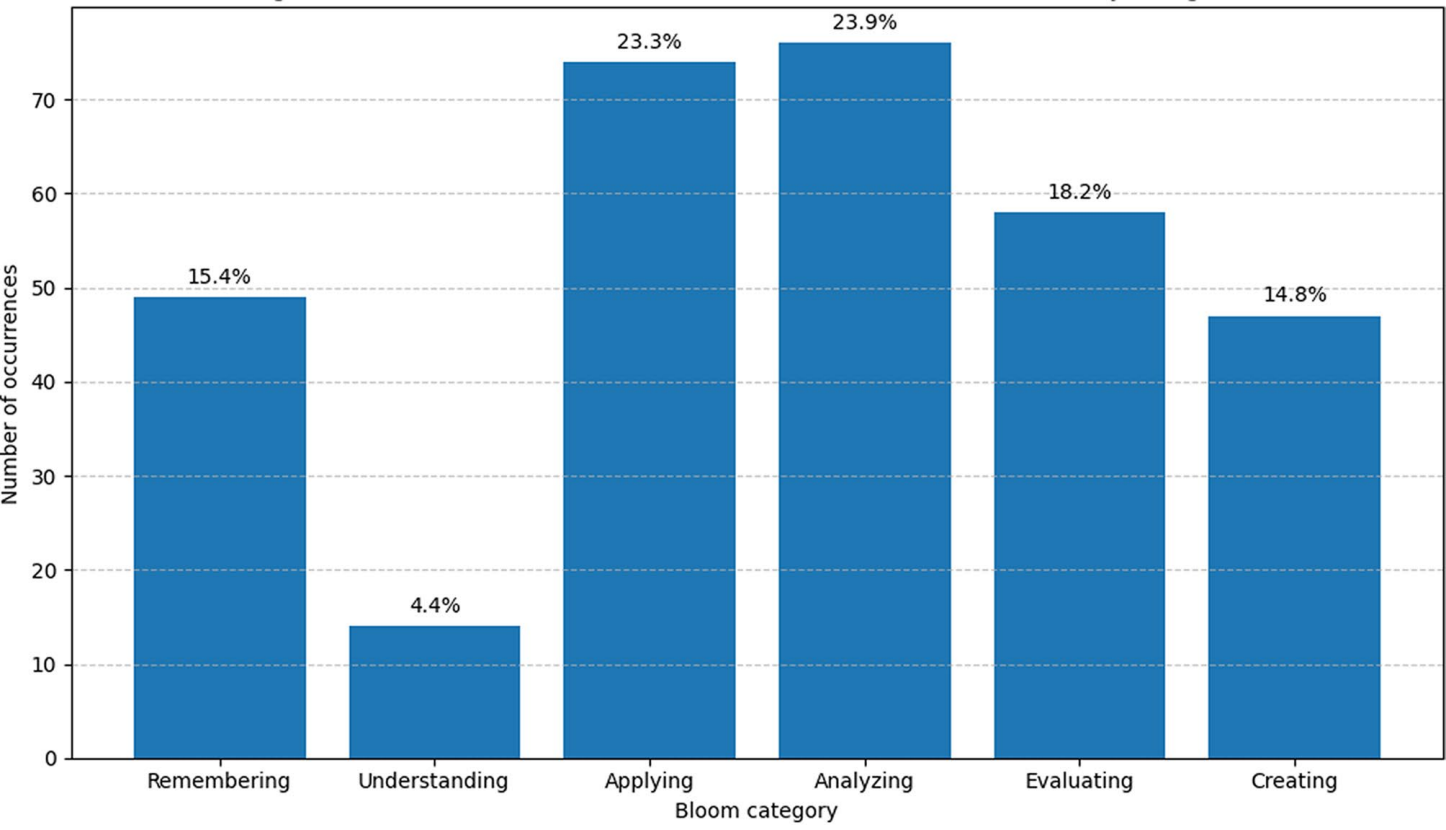
Distribution of active verbs across Bloom’s revised taxonomy categories

Figure 1 illustrates the frequency and proportional distribution of active verbs across Bloom’s six taxonomy categories, emphasizing the predominance of analyzing and applying active verbs in the sample.

### Taxonomic breadth in active verb use

All syllabi employed active verbs from at least four of the six Bloom categories. Two institutions (8.7%) used active verbs from exactly four categories, twelve (52.2%) from five categories, and nine (39.1%) from all 6. The number of Bloom categories represented per syllabus showed a weak and non-significant correlation with the proportion of higher-order active verbs (ρ = −0.20, *p* = 0.366), suggesting that greater taxonomic breadth does not equate to greater cognitive complexity. Table 3 presents the cognitive distribution of active verbs, categorized according to Bloom’s revised taxonomy, for each institution. To further assess the structural alignment of syllabi with the Swedish Higher Education Ordinance, analyses were restricted to the 175 learning outcomes derived from the 17 syllabi that explicitly employed this tripartite framework. These outcomes were categorized into the domains of knowledge and understanding, competence and skills, and judgement and approach, allowing for domain-specific cognitive analysis.

**Table 3:** Cognitive distribution of active verbs by Bloom’s revised taxonomy per institution

Higher Education Institution	Remembering	Understanding	Applying	Analyzing	Evaluating	Creating	Total number of active verbs	Number of unique active verbs	Number of Bloom’s revised taxonomy levels represented	Proportion of higher-order* cognitive verbs (%)
Blekinge Institute of Technology	4	2	2	4	5	2	31	19	6	35,5
University of Borås	3	1	7	3	3	2	22	19	6	36,4
Dalarna University	2	1	4	4	2	2	21	16	6	38,1
University of Gävle	2	1	3	4	3	1	22	14	6	36,4
Halmstad University	2	1	4	2	0	2	16	11	5	25,0
University of Skövde	2	1	4	4	3	2	22	16	6	40,9
University West	4	1	3	4	2	1	20	16	6	35,0
Marie Cederschiöld University College	1	2	2	2	3	0	12	10	5	41,7
Red Cross University College	3	0	3	2	1	4	17	13	5	41,2
Sophiahemmet University College	3	0	3	5	3	3	23	16	5	47,8
University of Gothenburg	2	0	3	4	2	1	18	11	5	38,9
Jönköping University	2	0	4	2	2	1	20	11	5	25,0
Karolinska Institute	2	0	3	1	2	2	16	10	5	31,3
Karlstad University	1	0	3	2	3	0	16	10	4	31,3
Linköping University	1	0	2	6	3	3	25	14	5	48,0
Linnaeus University	1	1	4	5	3	3	24	17	6	45,8
Luleå University of Technology	4	1	4	3	2	3	21	16	6	38,1
Lund University	3	0	0	2	3	4	14	12	4	64,3
Mälardalen University	1	0	2	4	3	4	15	14	5	73,3
Mid Sweden University	1	2	4	2	3	2	24	12	6	29,2
Umeå University	2	0	1	3	4	3	14	13	5	71,4
Uppsala University	1	0	5	4	1	1	14	12	5	42,9
Örebro University	2	0	4	4	2	1	22	13	5	31,8
	**49**	**14**	**74**	**76**	**58**	**47**	**449**	**315**		

*Analyze, Evaluate, Create

### Alignment with national learning outcome categories

Our research question focused on how the learning outcomes are distributed across the three national domains of knowledge. We found that seventeen HEIs provided syllabi explicitly structured according to the ordinance’s three learning outcome categories. This analysis was based on a defined subset (*n* = 17) of the full sample, thereby enhancing internal consistency and enabling dimension-specific interpretation. The 172 learning outcomes extracted from these 17 syllabi were distributed across the three national framework domains as follows: 25% addressed Knowledge and Understanding (*n* = 33), 46% targeted Competence and Skills (*n* = 61), and 29% addressed Judgement and Approach (*n* = 39). This distribution is further illustrated in Fig. 1, which presents the relative emphasis placed on each framework domain across institutions employing structured syllabi. Table 4 complements this figure by presenting the exact number and percentage of learning outcomes and active verbs per ordinance category, based on structured syllabi only.

**Table 4:** Distribution of learning outcomes and active verbs by ordinance category (structured syllabi only)

Higher Education Institution	Total number of learning outcomes	*n* learning goals in KU*	Proportion of verbs in KU*(%)	*n* learning goals in SA**	Proportion of verbs in SA** (%)	*n*learning goals in JA***	Proportion of verbs in JA*** (%)
Blekinge Institute of Technology	17	3	18	7	41	7	41
University of Borås	11	3	27	6	55	2	18
Dalarna University	11	-	-		-		-
University of Gävle	11	-	-		-		-
Halmstad University	8	2	25	3	38	3	38
University of Skövde	10	3	30	4	40	3	30
University West	12	3	25	5	42	4	33
Marie Cederschiöld University Colleg	9	2	22	5	56	2	22
Red Cross University College	10	2	20	5	50	3	30
Sophiahemmet University College	12	2	17	6	50	4	33
University of Gothenburg	10	5	50	3	30	2	20
Jönköping University	7	1	14	4	57	2	29
Karolinska Institute	10	3	30	4	40	3	30
Karlstad University	7	1	14	5	71	1	14
Linköping University	13	4	31	5	38	4	41
Linnaeus University	10	-	-		-		-
Luleå University of Technology	12	3	25	7	58	2	17
Lund University	9	3	33	3	33	3	33
Mälardalen University	11	-	-		-		-
Mid Sweden University	10	-	-		-		-
Umeå University	7	2	29	2	29	3	43
Uppsala University	8	2	25	4	50	2	25
Örebro University	8	-	-		-		-
	**233**	**44**		**78**		**50**	

*Knowledge and Understanding (KU); **Skills and Abilities (SA); ***Judgement and Approach (JA)

**Fig. 2:**
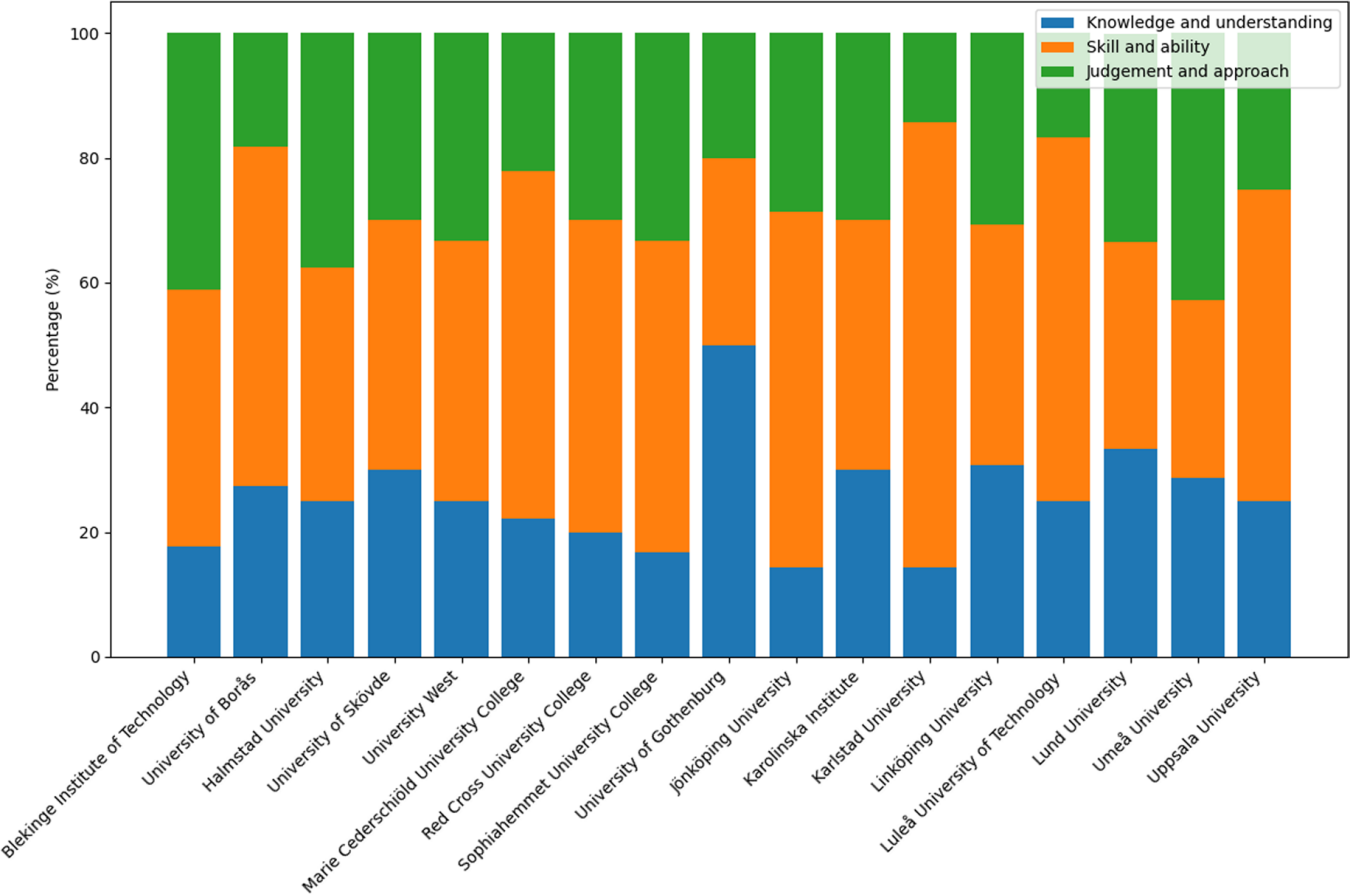
Proportion of intended learning outcomes by ordinance category

Figure 2 visualizes the relative proportions of learning outcomes across the three ordinance dimensions among the structured syllabi.

**Fig. 3:**
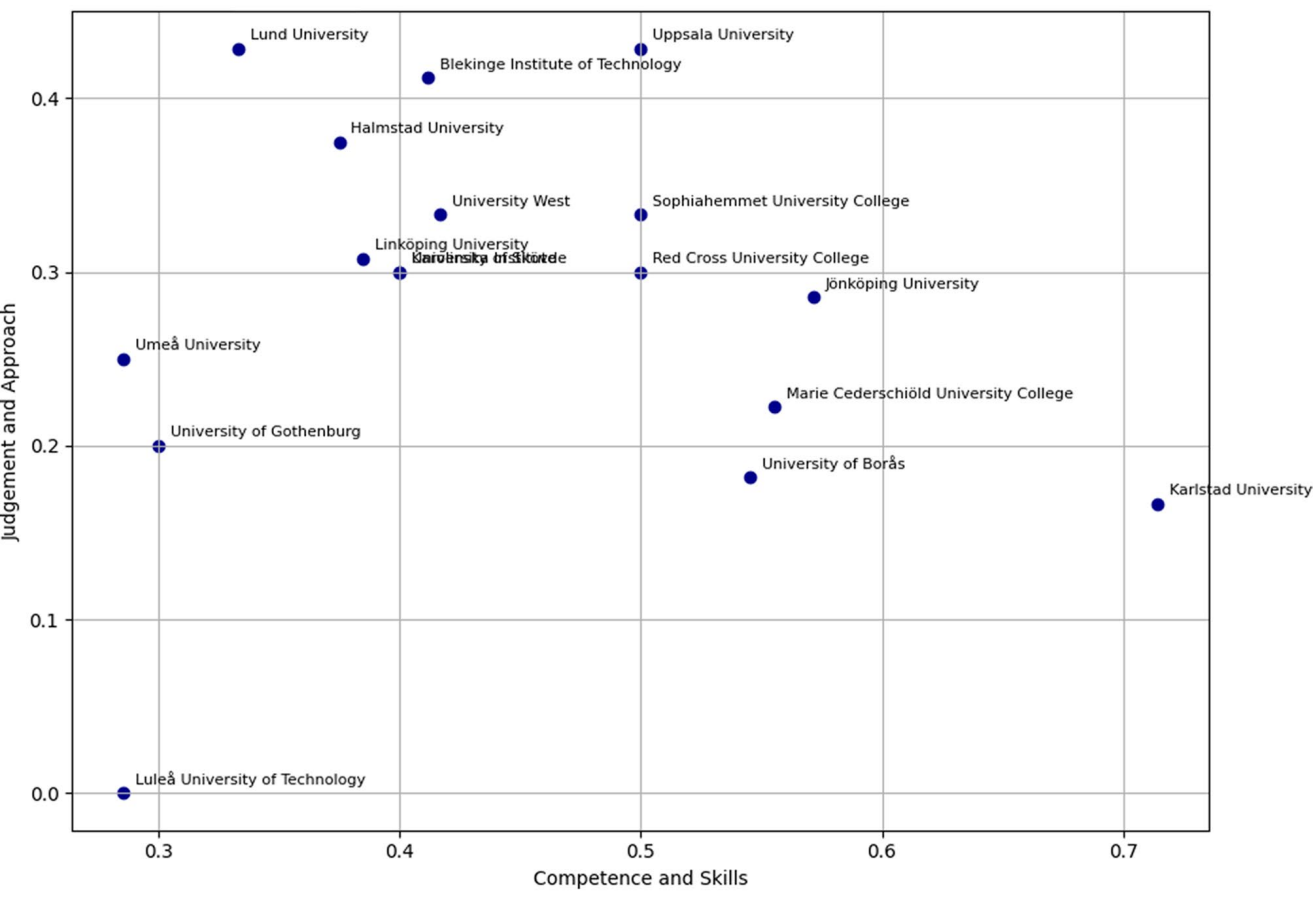
Typology of learning outcome distribution across HEIs (2D)

Figure 3 presents this mapping, illustrating how institutions cluster in terms of outcome emphasis.

A total of 455 active verb occurrences were identified across all syllabi. The most frequent Bloom categories were *Analyzing* (*n* = 114, 25.1%) and *Applying* (*n* = 111, 24.4%), followed by *Evaluating* (*n* = 80, 17.6%). *Understanding* was the least frequent category (*n* = 15, 3.3%). These patterns are further illustrated in Fig. 2, which shows the absolute and relative frequencies of active verbs distributed across Bloom’s six taxonomy categories. Of the 58 unique active verbs identified, only 20 (34.5%) matched those explicitly listed in Bloom’s revised taxonomy; the remainder were categorized through interpretive judgement and expert consensus. Table 5 provides the numeric breakdown of active verb frequencies and diversity across Bloom’s six taxonomy levels. Figure 3 presents a two-dimensional typology, mapping institutions’ emphasis across the Competence and Skills and Judgement and Approach dimensions.

**Table 5:** Frequency and diversity of active verbs per Bloom category

Bloom category	Number of occurrences	Proportion (%)	Number of unique verbs
Remembering	71	15,6%	6
Understanding	15	3,3%	6
Applying	111	24,4%	14
Analyzing	114	25,1%	10
Evaluating	80	17,6%	11
Creating	64	14,1%	11

## Discussion

This study aimed to examine the structure, cognitive complexity, and knowledge domain classification of learning outcomes in degree project courses within Swedish undergraduate nursing education. The findings show that higher-order thinking is not uniformly emphasized across institutions. Instead, a variety of positions and interpretations emerge concerning the formulation of active verbs and learning objectives. Despite this variation, a shared alignment with national degree objectives is consistent.

These patterns can be interpreted as an indication that the content in the courses is strongly institution-driven, with many institutions developing their course plans independently, without benchmarking against others. This institutional diversity can be viewed from two perspectives. On one hand, it may lead to inconsistencies in educational quality and graduate outcomes. On the other hand, it reflects a rich variety of educational approaches that foster innovation and adaptability. Such variation aligns with Fink’s [6] model of significant learning experiences, which emphasizes the importance of contextually tailored course design to create meaningful and lasting learning. Similarly, Anderson and Krathwohl’s [5] revised taxonomy provides a useful framework for understanding how different institutions prioritize and structure cognitive processes and knowledge domains within their learning outcomes. However, the uneven emphasis on higher-order cognitive skills also points to a need for more deliberate scaffolding of learning objectives to ensure progression towards complex thinking, as advocated in educational research on cognitive development. Future curriculum development might benefit from balancing institutional autonomy with shared benchmarks to enhance both innovation and consistency in nursing education. We read Swedish thesis policy through a paradox lens [29]: the same regulatory scripts that secure minimum quality simultaneously threaten curricular experimentation. Institutions respond through ‘selective coupling’ [30], adopting the vocabulary of UKÄ audits while locally bending it to distinctive pedagogical niches—hence the verb diversity we found. Such maneuvering illustrates how innovation and assurance are not opposing poles but inter-dependent logics that must be continuously balanced. Recognizing this paradox invites stakeholders to evaluate thesis courses not only against compliance metrics but also against their capacity to generate novel, context-responsive forms of knowing.

Sweden’s seemingly uniform emphasis on higher-order verbs across both universities and university colleges, despite stark asymmetries in research funding and staff densities, can be read as a textbook case of institutional isomorphism (DiMaggio & Powell 1983). Coercive forces emanate from the Higher Education Ordinance [19] and the UKÄ quality-assurance cycle [24]; failure to comply jeopardizes accreditation. Normative pressures flow from the Bologna agenda and from professional bodies that equate the bachelor thesis with scientific legitimacy, while mimetic impulses encourage less-resourced colleges to copy the rhetorical templates of flagship universities. These overlapping logics flatten local variation, explaining why our Mann–Whitney U tests detected no significant university–college differences.

Drawing on Aristotle’s tripartite schema—*epistēmē* (scientific knowing), *technē* (rule-based craft) and *phronēsis* (situated judgement)—and on Bornemark’s [31] critique of measurability rationality, we argue that verb auditing privileges *technē*: verbs, credit points and audit trails are readily quantified and therefore feed a bureaucratic apparatus that expands irrespective of whether curricula actually cultivate *epistēmē* or *phronēsis*. Into this discourse enters the Moderate Party which just recently proposed to omit the thesis and shorten nursing, social-work and police programs by up to one semester [25]. The reform is framed as a solution to workforce shortages and student debt, not as a swap for alternative pedagogies. Our data suggest that eliminating the thesis would further tilt the curriculum toward routinised *technē*, potentially hollowing out both *epistēmē* and *phronēsis*.

Multiple studies [32–34] reveal that the degree project course in nursing is far from being a mere procedural requirement. It constitutes a pivotal site of epistemic engagement, namely, students’ active, norm-guided participation in producing and evaluating knowledge claims [35], and professional formation. Therefore, it is advantageous that, according to the present study, the same conditions for this appear to exist throughout Sweden. This is not the case internationally. Rocca et al. [34], reviewing 66 Spanish nursing curricula, identified significant variation in ECTS weighting (ranging from 6 to 12 ECTS), the number and type of competencies, and the presence of learning outcomes—only 40.9% of programs included explicit intended learning outcomes. A wide disparity in the formulation of competencies (generic, general, and specific) suggests a lack of consensus across institutions.

The widespread use of active verbs from at least four of the six Bloom’s revised taxonomy [5] categories across all syllabi suggests a broad and varied approach to cognitive skill development in nursing education. Yet even Anderson & Krathwohl’s [5] revised Bloom supplies only a *partial* map of cognitive complexity. Its linear ladder foregrounds knowledge processing but sidelines socio-affective growth and integrative learning [36]. Recent nursing work [37] systematically aligned an undergraduate evidence-based-practice course with Fink’s six dimensions demonstrating that such design fosters self-directed, ethically grounded and equity-attentive learning—outcomes that lie beyond Bloom’s cognitive spine and therefore merit future parallel scrutiny in Swedish syllabi.

As shown in Fig. 1, the learning objective of understanding consistently has a low presence in the curricula across all institutions, whereas the objective of memorization is surprisingly more prevalent. This observation is noteworthy because achieving higher cognitive levels and higher-order thinking is challenging without adequate training in understanding. The limited emphasis on understanding in these curricula is difficult to explain but may be attributed to several factors, such as efforts to avoid overloaded curricula or assumptions that understanding will be addressed in other courses. One way to interpret this is that previous theoretical understanding is expected to be translated into step-by-step knowledge of how academic knowledge production should be carried out. This may reflect a mechanical view of learning, where technē, methodical craft and practical know-how is prioritized over epistēmē, a deeper theoretical insight. Such an approach aligns with a routinised pedagogy, where learning outcomes are formulated to allow for easier control and assessment. Memorization thus becomes an attractive alternative, as it is simpler to measure than broader academic understanding, again with a risk of undermining the development of situated judgement (phronēsis) that is essential in nursing practice. To address this, pedagogical changes could include teaching methods that foster student-led, active engagement with course content and emphasize real-life experiences and reflective seminars. For example, Jordal et al. [23] highlight how structured opportunities for students to compare, reframe, and apply knowledge to new contexts in authentic learning activities can foster responsibility, autonomy, and deeper conceptual understanding.

The design of learning outcomes not only matters for transparency in assessment and how the thesis is actually evaluated but also has significance for the approach or design that students choose. Several studies indicate that nursing thesis are overwhelmingly qualitative, often grounded in phenomenological or inductive content-analytic traditions [34]. While pedagogically legitimate, this methodological pattern raises questions about how learning outcomes may preconfigure the range of epistemic options students perceive as available. If active verbs associated with comprehension and description dominate syllabi, the taxonomic signaling may inadvertently steer students away from experimental, mixed-methods, or theory-driven designs. This phenomenon has international resonance. Scholars have emphasize how clinical immersion and reflective practice shape the contours of professional nursing identity [38, 39]. However, they also identify a recurring tension between pedagogical aspiration and curricular implementation. Similarly, Garner et al. [40] show how sociocultural dynamics constrain student engagement, reinforcing the imperative for learning outcomes that are both cognitively ambitious and contextually attuned.

Karlsholm et al. [41] further emphasize how social and individual contexts shape students’ writing trajectories. Their study foregrounds the difficulties students encounter in mastering research methodology, especially in empirical projects, and identifies diverse coping strategies—ranging from academic writing routines to informal peer support and varied supervisory experiences. Taken together, this portrays the thesis course not only as a space for producing academic work but also as an arena for navigating uncertainty, identity, and professional transformation. These insights indicate that the formulation of learning outcomes in thesis courses is not a neutral exercise in curricular taxonomy but a decisive act that frames the interpretive horizon of the student’s final academic undertaking. Learning outcomes that privilege lower-order active verbs may unwittingly limit the scope of methodological exploration and conceptual risk-taking, nudging students toward descriptive rather than analytical or generative engagements. Therefore, it is crucial to carefully consider how learning outcomes are formulated to ensure they support both deep learning and methodological innovation in thesis projects. This function of the thesis as a gateway to research engagement is reinforced by Dogan et al. [15], whose scoping review found that writing a bachelor’s thesis positively shaped students’ attitudes toward research and evidence-based practice.

Within Biggs’ constructive alignment model, intended learning outcomes form one leg of a triad—outcomes, teaching/learning activities, and assessment—that must be mutually reinforcing to optimize learning [9]. By mapping the cognitive level of thesis outcomes, our study analyses this first node, providing an empirical baseline against which future work can evaluate whether Swedish nursing programs achieve full alignment across teaching and assessment. Other research has also highlighted the importance of the design of learning outcomes beyond being purely administrative tools. A pivotal study that has significantly influenced the conceptualization of higher-order thinking and thesis work in nursing education is the research conducted by Kapborg and Berterö [42]. Their study addresses the challenge of evaluating Bachelor candidates’ thesis in relation to learning objectives. By establishing and endorsing the use of checklists for assessing student learning outcomes, their work has fostered a checklist mentality that has since dominated nursing faculty assessment discussions, often overshadowing the complex qualitative and epistemological dimensions of curricula content [42]. Wickström [43] critically expands on this issue, emphasizing that a checklist mentality risks reducing educational complexity and teacher-student agency. This reduction tends to favor methodical craft and procedural competence (technē) while marginalizing a deeper theoretical understanding (epistēmē), that underpins academic inquiry that also may hinder the cultivation of phronēsis. Wickström [43] instead argues for educational models that acknowledge disciplinary differences and foster deeper, epistemologically grounded discussions about student learning outcomes, moving beyond purely technical or bureaucratic approaches.

In a similar vein, Jordal et al. [23] present a supervising model which emphasizes the need for structured yet dialogical supervision that supports students’ autonomy and critical academic engagement. The model shifts the focus from directive feedback toward co-constructive reflection, aligning with the broader goal of fostering reasoning, communication, and independent scholarly development. Our study reinforces these perspectives, showing that despite national regulatory frameworks creating considerable uniformity, the diversity in active verb usage and cognitive complexity embedded in thesis-course syllabi indicates underlying tensions between standardized assessment practices and the pedagogical intent to foster genuine higher-order thinking. This highlights the need to critically examine whether current assessment practices truly align with epistemologically ambitious curricular aims or inadvertently perpetuate a restrictive approach to student learning. We encourage colleagues to analyze these aspects in nursing thesis in future research. These results should be read through the lens of constructive alignment: we have documented the “intended outcome” component, but whether teaching activities and assessment tasks in thesis courses are calibrated to the same cognitive levels remains an open question for future research.

### Implication for practice

In a metrics-driven quality system, credits, verb tallies, and audit trails can let technē eclipse both epistēmē and phronēsis. Thesis syllabi must therefore be forged in collegial dialogue, led by faculty with deep pedagogical expertise, so their learning outcomes consciously braid scientific inquiry (epistēmē), methodical craft (technē), and situated judgement (phronēsis). Through constructive alignment, teaching and assessment translate those aims into practice, guiding programs—and students—from exploratory searching, through purposeful aiming, toward sound academic judgement. Supervisor development that favors phronetic conversation over checklist compliance powers this journey, anchoring accountability in the learner’s genuine quest for knowledge and keeping the bachelor thesis a formative professional rite rather than a bureaucratic ritual.

### Methodological considerations

Several methodological strengths enhance the rigor of this study. First, data collection was comprehensive, encompassing all available syllabi from nursing programs in Sweden. A systematic and transparent extraction process ensured consistent representation across institutions. Active verbs were identified, semantically reviewed, and linguistically consolidated through collaborative validation, which reduced subjective bias and strengthened theoretical coherence. Bloom’s revised taxonomy [5] served as the analytical framework, and active verbs were independently classified and cross-validated among researchers to ensure consistency. Although inter-rater reliability was not quantified statistically, peer review helped mitigate coder subjectivity. Descriptive and inferential statistics were conducted using Excel, supported by Python for exact p-values. Spearman’s rank correlation coefficients were used to assess monotonic associations, appropriate given the small sample size (*n* = 23) and non-parametric nature of the data [27]. All computations were performed manually and documented stepwise to ensure transparency and reproducibility. A noted limitation was the decision not to quantify the number of active verbs per individual learning outcome. Although analytically feasible, this was beyond the study’s scope. Nonetheless, the analysis included measures of verb redundancy and lexical diversity, offering insight into the variation in cognitive emphasis across institutions. Finally, our study interrogates documents rather than lived practice; we did not observe classroom interactions or gather student perspectives. Consequently, the cognitive level inferred from verbs may diverge from the complexity enacted in teaching and assessment. Future mixed-methods work should triangulate syllabi with observational and learner-experience data to test this ‘implementation gap’.

## Conclusions

This study demonstrates that, while nursing programs in Sweden align with national standards, they vary in how they embed higher-order thinking within thesis-course learning outcomes. Although active verb usage appears relatively consistent, the cognitive depth and epistemic orientation of these outcomes differ, reflecting distinct pedagogical priorities. The thesis course thus serves as a critical space for professional development and epistemic engagement, shaped by institutional context and supervisory practice. Future research should examine how learning outcome formulation interacts with supervision, methodology choice, and student learning, to support more coherent and epistemologically grounded curriculum design in undergraduate nursing education.

## Data Availability

No datasets were generated or analysed during the current study.

## Declarations

### AI declaration

Use of large language models (LLMs) in statistical calculations and visualization is described in Sect. “Use of AI tools for statistical calculations and visualization”. During multiple stages of this study ChatGPT-4o, ChatGPT-4.5, ChatGPT-o1 (April 2025), was employed as a coding assistant to develop Python scripts for data processing and visualization. Code was generated iteratively in collaboration with the authors via Google Colab, and targeted tasks included cleaning and structuring Excel-derived datasets, computing descriptive and inferential statistics (e.g., Spearman’s ρ), and rendering figures (e.g., Bloom category distributions, institutional typologies). All AI-generated code was manually reviewed, edited, and executed by the authors to ensure computational validity, reproducibility, and alignment with the study’s analytical aims. No statistical inference or interpretive output was directly accepted without verification. Additionally, ChatGPT was used for linguistic polishing, reformulation and reorganization of paragraphs for clarity, and condensation of dense sections, without altering the original argumentation, epistemic stance, or analytical conclusions. The final manuscript reflects the authors’ conceptual design, analytic decisions, and scholarly interpretation. All AI contributions were non-autonomous and subject to critical human oversight.

## Abbreviations

HEIs: Higher Education Institutions
ECTS: European Credit Transfer System
